# Well controlled maternal inflammatory bowel disease does not increase the risk of abnormal neurocognitive outcome screening in offspring

**DOI:** 10.1016/j.bbih.2024.100827

**Published:** 2024-07-22

**Authors:** Ralley E. Prentice, Rod W. Hunt, Alicia J. Spittle, Michael Ditchfield, Jeff Chen, Megan Burns, Emma K. Flanagan, Emily Wright, Alyson L. Ross, Rimma Goldberg, Sally J. Bell

**Affiliations:** a Department of Gastroenterology, Monash Health, Melbourne, VIC, Australia; b Department of Gastroenterology, St Vincent's Hospital Melbourne, Melbourne, VIC, Australia; c Department of Medicine, Monash University, Melbourne, VIC, Australia; d Department of Neonatal Medicine, Monash Health, Melbourne, VIC, Australia; e Department of Paediatrics, Monash University, Melbourne, VIC, Australia; f Cerebral Palsy Alliance, Australia; g Department of Physiotherapy, University of Melbourne, Melbourne, VIC, Australia; h Victorian Infant Brain Studies, Murdoch Children's Research Institute, Melbourne, VIC, Australia; i Department of Medical Imaging, Monash Children's Hospital, Melbourne, VIC, Australia; j Department of Medicine, University of Melbourne, Melbourne, VIC, Australia

**Keywords:** Inflammatory bowel disease, Pregnancy, Cerebral palsy, BabyMoves, Generalised movement assessment

## Abstract

**Background:**

Exposure to maternal inflammation is associated with an increased risk of neurocognitive and developmental disorders in offspring. Early diagnosis and intervention improves childhood motor and cognitive functioning. Neonatal cerebral MRI and remote app-based generalised movement assessments (GMAs) are both predictive of adverse neurocognitive outcomes but have only been used in infants at significantly increased risk for these outcomes, rather than following in utero exposure to maternal inflammatory disorders.

**Methods:**

Pregnant women with inflammatory bowel disease were assessed clinically and biochemically in each trimester of pregnancy in this single centre prospective study. Neonatal cerebral MRIs were performed at 6–12 weeks post-corrected term. Two GMA videos were filmed using the ‘BabyMoves’ app from 12 to 16 weeks of age. MRIs and GMAs were assessed by a blinded highly qualified practitioner using validated scoring systems.

**Results:**

40/53 of invited maternal-infant dyads were recruited. C-reactive protein was elevated antenatally in less than 13%. 5/37 neonatal MRIs had incidental or obstetric trauma related gross anatomical abnormalities, with none abnormal on validated gross abnormality scoring. 3/35 GMAs were abnormal, with one GMA abnormality being clinically significant. Of those with abnormal GMAs, 2/3 were in exposed to severely active IBD in-utero.

**Conclusion:**

Neonatal cerebral MRI and GMA for neurocognitive screening is feasible in the setting of maternal inflammatory bowel disease, where the risk of cerebral palsy is poorly defined and thus burdensome screening interventions are less appealing to parents. Larger studies are required to stratify adverse neurocognitive outcome risk in infants born to women with maternal inflammatory disorders, but these data are reassuring for women with IBD in remission antenatally.

## 1 Introduction

Inflammatory bowel disease (IBD), comprising Crohn's Disease (CD) and Ulcerative colitis (UC), are relapsing and remitting inflammatory disorders of the gastroenterological tract. The prevalence of IBD is between 200 and 500 per 100,000 in developing countries, with an increasing incidence and disease burden globally (Ng et al., 2017). These conditions commonly affect women of childbearing age, with active disease impacting between 20 and 50% of pregnancies (Vestergaard et al., 2023; Meyer et al., 2020).

A single large registry based cohort study has identified an increased risk of cerebral palsy in the offspring of women with Crohn's disease (Strøm et al., 2021), although to date, an increased risk of developmental abnormalities has not been observed in prospective cohort and relatively small registry-based cohort studies (Mahadevan et al., 2021; Friedman et al., 2020). However, the validity of the latter findings are debatable given an infrequent exposure and outcome, with a lack of non-IBD control cohort in prospective studies.

Specific cerebral magnetic resonance imaging (MRI) sequences can now assess the structural integrity of the neonatal brain, generating a ‘gross abnormality score’ that serves as an accurate surrogate marker for early motor and working memory deficiencies and later neurocognitive outcomes including diagnosis of cerebral palsy and autism spectrum disorder (Anderson et al., 2017; Ciarrusta et al., 2020; Cheong et al., 2012; Woodward et al., 2006). Moreover, simple measurements of brain size, particularly brain biparietal diameter, correlate with cognitive and motor indices at 2 years of age in extremely preterm infants (Tich et al., 2011).

Generalised spontaneous movements, which are observed constantly in all infants between birth and 20 weeks post term age, tend to lose their variability and complexity and become excessively jerky in infants with neurological abnormalities (Prechtl, 2001; Phagava et al., 2008). Formalised general movements assessments can be used in infants for early cerebral palsy screening, with a high sensitivity for cerebral palsy diagnosis, particularly in conjunction with cerebral MRI (Novak et al., 2017). The validated ‘BabyMoves’ app allows parents to remotely record a video of their infant so that generalised movement assessments can be subsequently scored (Kwong et al., 2019; Spittle et al., 2016; Peyton et al., 2024)^-^.

Instituting childhood medical screening can be undermined by parental anxiety surrounding the perceived harm of the intervention to the child (Carlton et al., 2021). To date, cerebral MRI and 10.13039/100031116GMA screening has been predominantly trialled in infants known to be at high risk for adverse neurocognitive outcomes, where the benefits of screening (including early diagnosis, intervention and access to financial support) are readily appreciable to parents (Cerebral Palsy Alliance, 2023). In this study, we aimed to demonstrate the feasibility of cerebral MRI and app-based GMA screening in infants born to women with IBD, where the associated risk of adverse infant neurocognitive outcomes is less well defined. We also aimed to perform explorative analyses correlating abnormalities detected by MRI and GMA with the presence of active maternal IBD over the course of pregnancy.

## 2 Materials and methods

### 2.1 Participants

Mother-infant dyads were recruited to this multicentre prospective cohort study between June 2021 and January 2023. Participants were invited to participate by two investigators (RP and SB) who explained the rationale for the study, the MRI and GMA procedures, the potential implications and follow up pathway if an abnormality was identified, and the safety of neonatal cerebral MRI. This information was reinforced with a detailed written consent form. Recruited maternal participants all had confirmed IBD. Maternal participants were excluded if they experienced a severe infection in pregnancy, or if they had a chronic viral infection, primary immunodeficiency, an additional chronic inflammatory disorder, or underlying renal, cardiac or hepatic impairment. Infant participants were excluded if born at less than 34 weeks' gestation Maternal participants were reviewed in each trimester of pregnancy, with both clinical (Physician Global assessment (PGA)) and biochemical (C-reactive protein (CRP), faecal calprotectin) assessments. Active disease in each trimester was defined clinically as a PGA ≥1 and biochemically as a CRP ≥15 mg/L and/or a faecal calprotectin ≥100 μg/g. These biochemical definitions were used as the upper limit of normal for CRP in pregnancy is higher than in the non-pregnant state (15–20 mg/L) (Watts et al., 1991), and preliminary data from our group has shown a faecal calprotectin ≥100 μg/g is associated with adverse pregnancy outcomes (Prentice et al., 2022). Accordingly, this faecal calprotectin level serves as our preconception and antenatal therapeutic target, with medical therapy typically escalated antenatally to achieve this target. We additionally performed analyses using an faecal calprotectin ≥250 μg/g to signify active disease, given published associations of an faecal calprotectin ≥250 μg/g in pregnancy and an increased risk of preterm delivery (Tandon et al., 2020). Maternal demographic data and obstetric outcomes were obtained via medical review, review of medical records and/or Redcap™ based surveys. Preterm delivery was defined as delivery <37 weeks’ gestation, and low birth weight as <2500 g at delivery.

### 2.2 Neonatal cerebral MRIs

Cerebral MRIs were performed at Monash Children's hospital, Melbourne, Australia when the infant was 6–12 weeks post corrected term age, using a 1.5T MRI. T_1_-weighted and T2/proton density-weighted sequences were performed. The MRIs were conducted without sedation, with infants fed to sleep then swaddled in a weighted blanket to prevent excessive movement (Elgueta et al., 2022).

A validated standardised scoring system was used to assess cerebral MRIs by a highly experienced independent blinded assessor (RH) using a DICOM browser (Woodward et al., 2006; Kidokoro et al., 2013). This scoring system generates the ‘global abnormality score’, grading abnormalities from 0 to 4 in four anatomical domains (cerebral white matter, cortical grey matter, deep grey matter and cerebellar abnormality). The overall score ranges from normal (0–3), mildly (4–7), moderately (8–11) and severely abnormal (>12). Simple measurements of different brain structures, defined as ‘metrics’, were also calculated using conventional MRI sequences and scored as per previous publications (Tich et al., 2011), and corrections applied for post corrected term age. Given the variation in age post corrected term at which the MRIs were performed, metrics were corrected for infant age by dividing this value by the corrected post term age in weeks and multiplying by 40. We acknowledge that post-natal cerebral growth of different cerebral structures is neither linear nor consistent between regions, however in the absence of good postnatal growth data, we opted for this simple standardisation. This standardisation has not been validated previously. Any additional gross anatomical abnormalities were reported and prompted referral for formal paediatric neurological and physiotherapy assessment.

### 2.3 Generalised movements assessment

For remote assessment of generalised movements, the BabyMoves app (available https://apps.apple.com/au/app/baby-moves/id1020058117 © Murdoch Children's Research Institute, 2019) was downloaded onto parent's smart phones. The infants were filmed via the app in the supine position wearing only a nappy on two separate occasions (between 12 weeks and 13 weeks and 6 days post corrected term, and again between 14 weeks and 15 weeks 6 days post corrected term age). These 3-min videos were directly uploaded to a secure RedCap™ database held by the Murdoch Children's Research Institute. Each video was scored by a blinded independent GMA certified assessor and trainer (AS). The assessor examined the infant's small amplitude, spontaneous, variable movements in all four limbs, termed fidgety movements. Fidgety movements were categorised as normal, absent or abnormal, where the movements are of exaggerated amplitude, speed and jerkiness. Abnormal or absent GMAs and MRIs prompted referral for formal paediatric physiotherapy and neurological assessment.

### 2.4 Statistical analysis

Statistical analyses were undertaken using STATA 17.0. Descriptive statistics were used for maternal demographics and obstetric outcomes (frequencies, median, range/IQR). Given few abnormalities were detected, detailed qualitative data are presented (Table 3). Exploratory associations between corrected biparietal diameter (BPD), and markers of maternal inflammation in each trimester of pregnancy were examined with spearman coefficients and both univariate continuous and univariate binary logistic regression. This simple brain metric was chosen specifically given the previously demonstrated association between BPD and cognitive and motor outcomes at 2 years of age in extremely preterm infants (Tich et al., 2011). Fisher's exact test was used to assess associations between abnormal GMAs and the presence of active disease. A p value of less than 0.05 was considered statistically significant.

## 3 Results

### 3.1 Maternal demographics and disease activity

**Fig. 1:**
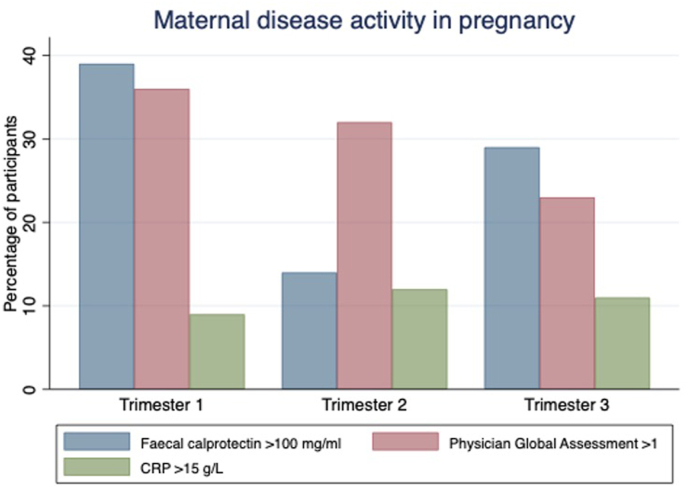
Maternal inflammatory bowel disease activity antenatally.

Of the 53 women invited to participate, 40 mother-baby pairs (75%) were recruited. Nineteen had Crohn's disease and 20 were exposed to a monoclonal biologic agent in pregnancy; 10 infliximab, seven adalimumab (both anti-tumour necrosis factor antibodies), two ustekinumab (an anti p40IL-12/23 antibody) and one vedolizumab (an α4β7 integrin inhibitor) (Table 1). 1/40 (2.5%) of women were current smokers and 3/40 (7.5%) consumed alcohol at volumes of less than two standard drinks per week in pregnancy. Disease activity was well controlled in most participants. Less than 13% had an elevated CRP, less than 40% had a faecal calprotectin more than 100 μg/g and 27.5% had a faecal calprotectin of more than 250 μg/g in pregnancy (Fig. 1). The median faecal calprotectin was 38.5 μg/g (IQR 13–227.5, n = 28), 22.1 μg/g (9.5–67.5, n = 36) and 37.6 μg/g (12.5–186, n = 34) in first, second and third trimester respectively, with the upper limit of normal in pregnancy being 50 μg/g (Julsgaard et al., 2017). Six participants were newly commenced on a biologic agent and/or received corticosteroid in pregnancy for management of active disease. Of those who required therapeutic escalation, only two did not subsequently achieve clinical and biochemical remission within six weeks. Of those with persistent disease activity, one had a mild elevation in faecal calprotectin requiring multiple courses of corticosteroid throughout pregnancy. The other required vedolizumab dose escalation, which was ineffective, so proceeded to infliximab induction in third trimester. One participant who required corticosteroids developed preeclampsia. No other corticosteroid related metabolic complications were observed. Eleven of the 24 women for whom data was available had COVID infection in pregnancy; one had COVID twice (in first and third trimester), three had COVID in first, four in second, and three in third trimester.

**Table 1:** Maternal participant disease and obstetric demographics.

	Maternal demographics			
Disease demographics	Age at start of pregnancy median (IQR) years			32 (29–36)
	Disease duration years (median/IQR)			(7.5) 2-20
	Crohn's disease (%) n/N	Total		(47.5) 19/40
		Perianal disease		(11) 2/19
		Prior intestinal resection		(37) 7/19
	Ulcerative colitis/IBD-unspecific (%) n/N	Total		(52.5) 21/40
	Current medication (%) n/N	Current thiopurine exposure		(27.5) 11/40
		Current Aminosalycylate use		(37.5) 15/40
		Current Biologic exposure (%) n/N	Infliximab	(25) 10/40
			Adalimumab	(18) 7/40
			Vedolizumab	(5) 1/40
			Ustekinumab	(5) 2/40
Obstetric history		Previous pregnancy (%) n/N		(62.5) 25/40
		Gravidity (median, range)		3 (1–8)
		Parity (median, range)		1 (0–3)
	Previous mode of delivery (%) n/N	Emergency c/section		(22) 4/18
		Elective c/section		(6) 1/18
		Vaginal		(56) 10/18
		Instrumental vaginal		(11) 2/18

### 3.2 Obstetric outcomes

The median gestational age, weight and length at delivery were 39 weeks (range 34 weeks and 2 days - 41 weeks), 3240gm (IQR 1793–4375) and 49 cm (IQR 47–50) respectively (Table 2). Over half of women delivered via caesarean section. As compared to the Australian population rates, the need for neonatal intensive or special care nursery admission was high, with 11/39 (28.2%) documented as being hypoxic or bradycardic at the time of delivery (Australian Institute of Health and Welfare, 2022a; Australian Institute of Health and Welfare, 2022b). Of those 12 infants requiring neonatal intensive care or special care nursery admission, seven were born to women with active biochemical disease (elevated CRP or faecal calprotectin) in pregnancy. With regards to additional risk factors for cerebral palsy, 2/39 (5.1%) and 3/30 (7.7%) were reported to have a fever or infection in labour, whilst 3/34 and 2/34 had an Apgar score ≤.7 at one and 5 min respectively.

**Table 2:** Frequency of obstetric outcomes in recruited pregnancies compared to those observed in the general Australian population From Australian Institute of Health and Welfare (2022) (Australian Institute of Health and Welfare, 2022a; Australian Institute of Health and Welfare, 2022b).

		Cohort		Australian population (%)
Gestational age at delivery (weeks, median & range)		39 (34+2–41)		
Preterm delivery (<37/40) (%) n/N		(5.0) 2/40		(8.2)
Birthweight (gm, median & range)		3240 (1793–4375)		
Low Birth Weight (<2500gm) (%) n/N		(10) 4/40		(6.3)
Birth length (cm, median & range)		49 (47–50)		
Congenital abnormalities (%) n/N		(5.0) 2/40		(3)
Need for NICU or SCN (%) n/N		(30) 12/40		(17)
Gestational diabetes (%) n/N		(7.5) 3/40		(16.3)
Gestation Hypertension (%) n/N		(10) 4/30		(3.2)
Preeclampsia (%) n/N		(5) 2/40		
In utero growth restriction^a^ (%) n/N		(20) 8/40		(20)
Post-partum hemorrhage (%) n/N		(2.5) 1/40		(1–5)
Apgar's (median, range)		1 min	9 (1–9)	
		5 min	9 (1–9)	
Apgar's≤7 (%) n/N		1 min	(8.8) 3/34	
		5 min	(5.9) 2/34	(2)
Mode of delivery (%) n/N	Emergency c/section	(22.5) 9/40		(38)
	Planned c/section	(30) 12/40		
	Vaginal	(37.5) 15/40		(50)
	Instrumental vaginal	(10) 4/40		(12.1)

a self-reported by participants.

### 3.3 Neonatal cerebral MRI

Overall, nine abnormalities were detected on either neonatal cerebral MRI or generalised movement assessments (Table 3). 37/39 MRIs were assessable; one was aborted at maternal request, and one was degraded by motion artefact. One participant failed to attend their MRI but later completed generalised movement assessments. The median corrected gestational age at the time of cerebral MRI was 46 weeks 6 days (range 42 weeks 5 days–53 weeks 4 days).

**Table 3:** Infants with abnormal neonatal MRI and Generalised Movement Assessments.

Maternal IBD history		Infant delivery			COVID in pregnancy	Abnormality		Clinical outcome	Antenatal maternal IBD activity
		Mode of delivery	Birth weight (grams)	Gestational age (weeks + days)		MRI	Generalised movement assessment		
1	UC. Subtotal colectomy with loop ileostomy	Normal vaginal	2400	37 + 5	No	Grey matter heteropia and dilated lateral ventricles	Normal	Nil.	Active disease T2 Peak CRP 20 g/L
2	L sided UC on 5ASA	Instrumental (forceps) vaginal	4280	40 + 4	No	Cerebellar microhaemorrhage	Normal	Nil	Active disease. Steroids from first to third trimester. IFX induction T3.
3	L sided UC on AZA	Normal vaginal with premature ROM	1790	34 + 2	Mild T3	Severe scaphocephaly	Normal	Nil	Remission
4	Pancolonic UC on 5ASA	Elective c/section	2619	37 + 1	Mild T2	Mild scaphocephaly	Normal	Nil	Active disease T3 Peak FC 407ug/g Normal CRP
5	Inflammatory colonic CD on AZA and IFX	Emergency c/section for obstructed labour with neonatal hypoxia	3920	39	No	White matter volume reduction	Normal	Nil	Remission
6	Penetrating ileal CD on AZA	Emergency c/section for preeclampsia	3960	38	No	Normal	Abnormal fidgety (only one video)	Declined f/up.	Active disease T1/2 IFX induction T2 Peak CRP 20 g/L
7	Inflammatory ileal CD on no medications	Elective c/section	2300	39.	No	Normal	Abnormal fidgety	Motor delay	Remission
8	L sided UC on no meds	Instrumental (forceps) vaginal	2840	40	Mild T2	Normal	Absent fidgety (only one video)	Nil	Active disease IFX induction T2 Peak FC 3840ug/g Normal CRP
9	L sided UC on 5ASA	Normal vaginal	3590	39	No	Normal	Abnormal hand flexion	Nil – resolved on follow up	Active disease T1 Peak FC 229ug/g Normal CRP

BW; birthweight, IFX; infliximab. T; Trimester. BM; Babymoves, AZA; azathioprine, 5ASA: aminosalycylate, UC; Ulcerative colitis. CD; Crohn's disease. CRP; C-reactive protein, ROM; Rupture of menbranes

Five macroscopic abnormalities were detected via neonatal cerebral MRI; cerebellar microhaemorrhages, an area of frontal grey matter heteropia, a large facial haemangioma and two cases of positional scaphocephaly. As per the review of a highly experience neonatal neurologist (RW), these abnormalities are likely to be incidental, related to prolonged neonatal intensive care unit admission or consequent to obstetric trauma (Table 3).

**Fig. 2:**
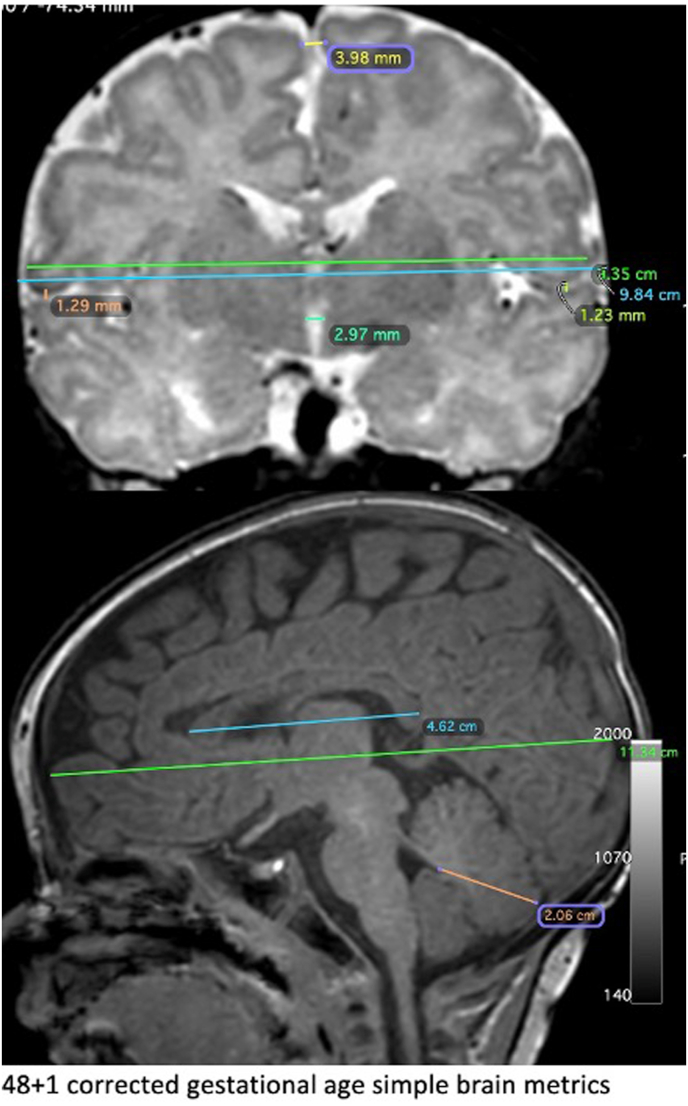
Example of a neonatal MRI with simple brain metrics measured.

All MRIs were normal when assessed by global abnormality score (Woodward et al., 2006; Kidokoro et al., 2013); 35/37 scored zero, 2/37 scored one. As such, associations between MRI global abnormality scores and generalised movement assessments, and MRI global abnormality scores and maternal CRP and faecal calprotectin, could not be assessed. With binary univariate logistic regression Elevated faecal calprotectin and CRP in trimesters one, two and three did not correlate with corrected biparietal diameter (Fig. 2). With continuous univariate logistic regression, Increasing faecal calprotectin and CRP at these timepoints was not associated with decreasing corrected biparietal diameter (Table 4).

**Table 4:** Spearman Correlation coefficients with corrected biparietal diameter and biochemical markers of disease activity antenatally. CRP; C-reactive protein, FC; Faecal calprotectin. For spearman correlation coefficients, elevated CRP defined as CRP >15 mg/L, elevated FC > 250 μg/mg.

		Trimester 1		Trimester 2		Trimester 3	
		CRP (n = 22)	FC (n = 27)	CRP (n = 31)	FC (n = 35)	CRP (n = 34)	FC (n = 33)
Corrected Biparietal diameter	Spearman correlation coefficient (r, p value)	0.08 P = 0.72	0.01 P = 0.95	0.24 p = 0.19	−0.05 p = 0.77	0.11 p = 0.52	0.08 P = 0.66
	Continuous inear regression coefficient (coefficient, 95% CI, p value	0.78 (−1.95-3.52) p = 0.056	−0.002 (−0.15-0.12) p = 0.82	0.057 (−0.63-0.75) p = 0.87	0.013 (−0.041-0.067) p = 0.631	0.009 (−1.16-1.18) p = 0.99	0.001 (−0.048-0.049) p = 0.982
	Linear binary regression coefficient (coefficient, 95% CI, p value)	14.98 (−38.14- 68.11) p = 0.56	−8.46 (−37.47-20.55) p = 0.55	−18.78 (−55.88- 18.32) p = 0.31	17.8 (−21.52-57.03) p = 0.36	7.91 (−27.84-43.66) p = 0.65	−1.65 (−30.37-27.06) p = 0.907

### 3.4 Generalised movement assessments

35/40 (87.5%) participants recorded at least one generalised movement assessment using the BabyMoves app, and all were of sufficient quality to be evaluated. Of these, 31/35 completed two generalised movement assessment s as directed. Three participants had abnormal generalised movement assessments (two abnormal fidgety, one absent fidgety). 2/3 of the infants with a abnormal generalised movement assessments were born to women with an elevated CRP in pregnancy (2/3 vs 4/32, p = 0.38 Fisher's exact) warranting commencement of infliximab in the second trimester. Of the nine women with a faecal calprotectin elevated above 250 μg/g in pregnancy, none had infants with an abnormal generalised movement assessment (9/32 vs 0/3, p = 0.53). None of the infants with abnormal generalised movement assessments were premature and none had a gross abnormality score >1 or macroscopic abnormality on cerebral MRI. One was of low birth weight (Table 3).

The parents of an infant with abnormal fidgety movements declined dedicated follow up, whilst the other received intervention for a delay in meeting motor milestones. The latter infant, born at term but of low birth weight to a mother with an uncomplicated pregnancy, was reviewed and deemed to be developing normally at five months of age. However, by 12 months of age, this infant was not sitting or crawling, in keeping with gross motor developmental delay. The infant with absent fidgety movements had only completed one GMA video and was meeting age-appropriate motor milestones on clinical review.

## 4 Discussion

This is the first study to utilise app-based generalised movement assessment tools combined with neonatal cerebral MRI to facilitate early assessment (and thereby intervention) for adverse neurodevelopmental outcomes in infants born to women with a chronic inflammatory disorder. An increased risk of brain microstructural abnormalities and abnormal generalised movements in those infants exposed to maternal IBD in-utero was not identified within the limitations of this small well-controlled cohort. Rather, we have shown that exposure to maternal IBD in remission is unlikely to result in adverse infant neurocognitive outcomes. Notably however, 2/3 abnormal GMAs were in the offspring of women with active disease and elevated markers of systemic inflammation.

Maternal inflammation in-utero, be that arising from infection or inflammatory disorders, has been associated with an increased risk of numerous adverse neurocognitive outcomes in offspring. In the aforementioned Norwegian registry study including over 1,360,000 children, the lifetime risk of cerebral palsy was increased in infants born to women with Crohn's disease (RR 2.1; 95% CI 1.0–4.1) and systemic lupus erythematosus (RR 2.7; 95% CI 0.9–8.3), including following adjustment for traditional risk factors for cerebral palsy (Strøm et al., 2021). Moreover, an Israeli cohort identified an increased risk of adverse neurodevelopmental outcomes in infants born to women following, as compared to infants born prior, to their mother's IBD diagnosis, although this observation is likely confounded by increasing maternal age (Dotan et al., 2013). The risk of Tourette's syndrome (adjusted incidence rate ratio 1.20; 95% CI 1.05–1.58) (Dalsgaard et al., 2015) and autism spectrum disorder (pooled odds ratio 1.34; 1.23–1.46) are also increased in the offspring of women with autoimmune disease (Chen et al., 2016; Han et al., 2021a). Likewise, an increased odds ratio for autism spectrum disorder has been observed in the offspring of women with bacterial infections in pregnancy (OR 1.13, 95% CI 1.03–1.23), being higher in those with more severe infections (OR = 1.30, 95% CI: 1.14–1.50) (Jiang et al., 2016). Certain viruses, such as cytomegalovirus, are known to be transmitted from mother to foetus in pregnancy, subsequently penetrating the foetal blood brain barrier and disrupting cerebral anatomical development (Elgueta et al., 2022). CMV infection in early pregnancy imparts an increased risk of sensorineural hearing loss and other neurodevelopment abnormalities including autism spectrum disorder (Adler et al., 2007; Maeyama et al., 2018). Non-neurotropic viral infections may also influence foetal neurodevelopment; despite not being shown to cross the placenta, antenatal maternal influenza A infection has also associated with an increased risk of schizophrenia and bipolar affective disorder in offspring (Elgueta et al., 2022).

The mechanism underlying the association between exposure to maternal inflammation in-utero and an increased risk of childhood neurodevelopmental disorders has been investigated in a number of animal and human studies, including in the setting of histological chorio-amnionitis, maternal systemic infection and maternal autoimmune disease. In murine models, changes in the abundance of innate and adaptive immune mediators, including TNF, interleukin (IL)-6, IL-17A, IL-1β and IL-2, in the foetal serum and thereafter at foetal cerebral neuronal synapses may compromise microglial and astrocyte function and synaptic plasticity (Strøm et al., 2021; Jiang et al., 2018; Han et al., 2021a, Han et al., 2021b; Mirabella et al., 2021; de Moura et al., 2021; Prieto-Villalobos et al., 2021; Potter et al., 2022; Estes and McAllister, 2016; Graham et al., 2018; Jain et al., 2022; Buchwald et al., 2022; Carbone et al., 2023; Tang et al., 2013; Zhang et al., 2019; Basil et al., 2014), with resultant persistent behavioural abnormalities in keeping with an autism spectrum phenotype (Strøm et al., 2021)^,^ (Jiang et al., 2018; Han et al., 2021a, Han et al., 2021c; Mirabella et al., 2021; de Moura et al., 2021; Prieto-Villalobos et al., 2021; Potter et al., 2022; Estes and McAllister, 2016; Graham et al., 2018; Jain et al., 2022; Buchwald et al., 2022; Carbone et al., 2023; Tang et al., 2013; Zhang et al., 2019; Basil et al., 2014; Malkova et al., 2012)^,^ (Humann et al., 2016)^,^ (Patterson, 2011; Choi et al., 2016; Kalish et al., 2021). For example, intraperitoneal injection of pregnant dams with poly(I:C) (designed to stimulate maternal systemic immune activation) results in an increase in the placental production of IL-17A, and thereafter an increase in IL-17RA mRNA expression in the foetal brain, resulting in disorganised cerebral lamination, and abnormal vocalisation, social recognition and repetitive behaviours in pups that are not observed following maternal treatment with an IL-17A blocking agent (Choi et al., 2016, Murakami et al., 2021). In human studies, an increase in cord blood pro-inflammatory cytokine abundance, including IL-1β, IL-6, IL-11, IL-13, IL-9 and TNF has been reported in infants with cerebral palsy versus healthy controls (Nelson et al., 1998), whilst infants born to women with elevated third trimester CRP, IL-6 and TNF have been shown to have lower cognitive ability and executive functioning as assessed by the Bayley Scales of Infant Development at six months and four years of age respectively (Camerota et al., 2022). Moreover, exposure to placental inflammation has been associated with an increased risk of abnormal mental development and psychomotor development scores at two years of age (Yanni et al., 2017).

An alternative mechanism by which maternal inflammation may predispose to abnormal neurodevelopment in offspring is via intestinal dysbiosis (Sun et al., 2023). Murine models have shown that pups born to antibiotic treated (‘dysbiotic’) dams have decreased thalamic axonal length, impaired somatosensory cortical function and abnormal tactile behavioural responses as compared to the offspring of untreated dams with a ‘normal’ intestinal microbiota (Vuong et al., 2020). In human cohort studies, higher maternal alpha-diversity in pregnancy has been associated with favourable infant behavioural outcomes at two years of age (Dawson et al., 2021), with women with IBD known to have lower alpha diversity in pregnancy as compared to non-IBD controls (Torres et al., 2020). In our cohort, IBD activity was relatively well controlled, particularly when considering systemic rather than localised intestinal (i.e. as indicated by faecal calprotectin) inflammation. Biological therapies, which have been shown to influence maternal cytokine levels in IBD pregnancies, were continued or commenced in over half of participants (Wu et al., 2022; van der Giessen et al., 2020). Accordingly, we were unlikely to observe significant abnormalities in neonatal cerebral MRI and generalised movement assessments attributable to an increase in maternal serum inflammatory mediators, although it is thought provoking that two of the three infants with abnormal generalised movement assessments were born to women with elevated CRP antenatally. It could be hypothesized that for adverse infant neurocognitive outcomes to be observed, the degree of antenatal systemic maternal inflammation must be substantial.

Most studies investigating the association between maternal antenatal inflammation and infant neurocognitive outcomes have relied on formal developmental assessments or parentally administered questionnaires, although the utility of neonatal cerebral MRI and generalised movement assessments have been demonstrated. For example, in healthy women, mean serum IL-6 concentration in pregnancy has been associated with abnormal connectivity of the fronto-limbic tracts on infant cerebral MRI at one and twelve months of age, with these tracts important in higher-order coordination and regulation of emotion. In addition, increasing maternal IL-6 in pregnancy is associated with increasing size of the infant's right amygdala and decreased impulse control at 2 years of age (Graham et al., 2018; Rasmussen et al., 2019; Rudolph et al., 2018). Infants exposed to maternal COVID-19, as well as the classic neurotropic TORCH infections (maternal toxoplasmosis, rubella, cytomegalovirus and herpes infections) in utero have been shown to have an increased risk of abnormal generalised movements (Fajardo et al., 2023; Aldrete-Cortez et al., 2022; Domagalska-Szopa et al., 2022). With our cohort, we have added to the growing literature supporting the use of these accurate, safe and relatively convenient screening tools for offspring neurodevelopmental disorders.

There are numerous limitations to this study. The small cohort size prevented exploration of associations between baseline demographic and medication exposures and neurocognitive outcomes. Maternal participants were not routinely tested for COVID throughout pregnancy, nor were they excluded from the study if non-severe COVD infection was reported. This represents a potential confounder. The use of only one blinded reviewer for both MRI and GMA assessments, and the wide age range at which the MRIs were performed, were additional limitations.

In conclusion, we have shown that screening investigations for adverse neurocognitive and developmental outcomes in the IBD population, where the risk of such outcomes is poorly defined (and thus the perceived risk-benefit of participation in screening is less favourable), is accepted by most parents. This establishes the basis for a larger controlled study including women with multiple inflammatory disorders, with associated cost-benefit analyses. Given the financial impact of cerebral palsy related disability exceeds $140,000 per person per year in Australia, demonstrating an overall benefit of expanded screening to include infants at moderately increased risk of adverse neurodevelopmental outcomes is plausible (Australian Institute of Health and Welfare, 2022b). By showing that the risk of adverse neurocognitive outcomes is unlikely to be increased in those with well controlled IBD, these data provide reassurance to individuals with IBD planning a pregnancy, and further reinforce the importance of achieving and maintaining antenatal IBD remission.

## Funding

This work was supported by a 10.13039/501100001779Monash University RTP scholarship to RP, by a 10.13039/501100000925National Health and Medical Research Council Postgraduate Scholarship to RP and by a Crohn's Colitis Australia Postgraduate Scholarship to RP.

## Ethics approval statement

Ethical approval for the study was obtained from the Human Research Ethics Committees at Monash Health (RES-20-739X) with adult participants consenting on behalf of their infants.

## CRediT authorship contribution statement

**Ralley E. Prentice:** Writing – review & editing, Writing – original draft, Formal analysis, Data curation, Conceptualization. **Rod W. Hunt:** Writing – review & editing, Supervision, Project administration, Methodology, Investigation, Formal analysis, Conceptualization. **Alicia J. Spittle:** Writing – review & editing, Methodology, Investigation, Formal analysis, Data curation, Conceptualization. **Michael Ditchfield:** Methodology, Data curation. **Jeff Chen:** Project administration. **Megan Burns:** Project administration. **Emma K. Flanagan:** Writing – review & editing, Supervision, Data curation. **Emily Wright:** Writing – review & editing, Supervision, Conceptualization. **Alyson L. Ross:** Project administration. **Rimma Goldberg:** Writing – review & editing, Supervision. **Sally J. Bell:** Writing – review & editing, Supervision, Project administration, Methodology, Investigation, Conceptualization.

## Declaration of competing interest

Alicia Spittle is a tutor with the General Movements Trust. Sally Bell has received consultation fees from AbbVie and Janssen, has received research grants for other investigator-driven studies/clinical trial funding from AbbVie, Janssen and Shire and has received speaker's fees from AbbVie and Janssen. Emily Wright has received speaker fees from Abbvie, Celltrion, Falk, Ferring, Janssen and Pfizer, consultancy fees from AbbVie and research support from AbbVie, Ferring and Janssen. The other authors disclose no conflicts.

## Data Availability

The authors do not have permission to share data.
